# Three-dimensional architecture of human diabetic peripheral nerves revealed by X-ray phase contrast holographic nanotomography

**DOI:** 10.1038/s41598-020-64430-5

**Published:** 2020-05-05

**Authors:** Lars B. Dahlin, Kristian R. Rix, Vedrana A. Dahl, Anders B. Dahl, Janus N. Jensen, Peter Cloetens, Alexandra Pacureanu, Simin Mohseni, Niels O. B. Thomsen, Martin Bech

**Affiliations:** 1 Department of Translational Medicine – Hand Surgery, Lund University, Jan Waldenströms gata 5, SE-205 02, Malmö, Sweden; 2 Department of Hand Surgery, Skåne University Hospital, Jan Waldenströms gata 5, SE-205 02, Malmö, Sweden; 3 Niels Bohr Institute, Copenhagen University, Blegdamsvej 17, 2100 Copenhagen, Denmark; 4 Department of Applied Mathematics and Computer Science, Technical University of Denmark, Richard Petersens Plads Building 324, 2800, Kgs Lyngby, Denmark; 5 ESRF, The European Synchrotron, 71 Avenue des Martyrs, 38000 Grenoble, France; 6 Department of Biomedical and Clinical Sciences, Linköping University, 581 83 Linköping, Sweden; 7 Department of Medical Radiation Physics, Clinical Sciences Lund, Lund University, 221 85 Lund, Sweden

**Keywords:** Peripheral neuropathies, Diabetes, Other nanotechnology

## Abstract

A deeper knowledge of the architecture of the peripheral nerve with three-dimensional (3D) imaging of the nerve tissue at the sub-cellular scale may contribute to unravel the pathophysiology of neuropathy. Here we demonstrate the feasibility of X-ray phase contrast holographic nanotomography to enable 3D imaging of nerves at high resolution, while covering a relatively large tissue volume. We show various subcomponents of human peripheral nerves in biopsies from patients with type 1 and 2 diabetes and in a healthy subject. Together with well-organized, parallel myelinated nerve fibres we show regenerative clusters with twisted nerve fibres, a sprouted axon from a node of Ranvier and other specific details. A novel 3D construction (with movie created) of a node of Ranvier with end segment of a degenerated axon and sprout of a regenerated one is captured. Many of these architectural elements are not described in the literature. Thus, X-ray phase contrast holographic nanotomography enables identifying specific morphological structures in 3D in peripheral nerve biopsies from a healthy subject and from patients with type 1 and 2 diabetes.

## Introduction

A variety of techniques are used to evaluate the macro- and microstructure in health and disease or following trauma of the peripheral nerve^1^. In clinical and experimental studies, the delicate structure of peripheral nerves can be visualized at various resolutions by conventional light and electron microscopy, generating two-dimensional (2D) images of thin tissue slices^2–4^, by laboratory source micro-CT^5–9^ and synchrotron X-ray phase contrast micro-CT for three-dimensional (3D) imaging^10–12^ and via other techniques^13–15^. To further understand dysfunction of the peripheral nervous system, a more detailed analysis is required to visualize the cellular network and subcomponents of the nerve trunk. As such, quantitative 3D structural data and images can be obtained by light or electron microscopical technique followed by alignment procedures, but such a procedure is difficult and time-consuming^16^. X-ray phase contrast holographic nanotomography, with a resolution in-between that of light and electron microscope, has recently been used to visualize myelinated nerve fibres and various subcomponents in sciatic nerves of healthy mice^17^ with isotropic voxel sizes below 100 nm. Furthermore, other advanced X-ray microscopical techniques have also been used to study teased myelinated nerve fibres from wild type mice and also to map out the element distribution in the myelin sheath (i.e. P, S, Cl, Na, K, Fe, Mn and Cu)^18^. However, such data is mainly restricted to a single isolated neuron. Human peripheral nerves have not previously been visualized by X-ray phase contrast holographic nanotomography.

Our aim was to evaluate the feasibility of using X-ray phase contrast holographic nanotomography to make a detailed map of the myelinated nerve fibres, including the nodes of Ranvier, and the intricate three-dimensional (3D) anatomical structures in human nerve biopsies from the upper extremity in a healthy subject and from two patients with type 1 and 2 diabetes.

## Methods

### Patients and nerve biopsies

From a previous study, on nerve biopsies from the posterior interosseous nerve^19,20^, we randomly selected, a healthy subject and patients with type 1 and 2 diabetes. According to our previously described technique^20^, a biopsy of the posterior interosseous nerve was harvested from the dorsal side of the distal forearm at the same time as the patient underwent carpal tunnel release^19,20^. The patients solely had carpal tunnel syndrome with compression of the median nerve, and no history of trauma to the posterior interosseous nerve. The nerve biopsies were immersion fixed as described earlier^21^ and post-fixed in osmium for visualization of the myelinated nerve fibres as well as embedded in Epon^21^. Post-fixation in osmium is not necessary for the present synchrotron technique, but the earlier published morphological analyses required such a procedure. From the previous study cohort, one sample from each patient category was selected; i.e. a male healthy subject (age span 55–60 years; present HbA1c 4.7%; 28.0 mol/mmol; nerve conduction study on the lower extremity showing no polyneuropathy), a female patient with type 1 diabetes (age span 50–55 years; duration of diabetes >40 years; present HbA1c 6.9%; 50.0 mol/mmol; nerve conduction study on the lower extremity showing polyneuropathy) and a female patient with type 2 diabetes (age span 60–65 years; duration of diabetes >10 years; present HbA1c 9.0%; 75.0 mol/mmol; nerve conduction study on the lower extremity showing no polyneuropathy).

### X-ray phase contrast holographic nanotomography

**Figure 1:**
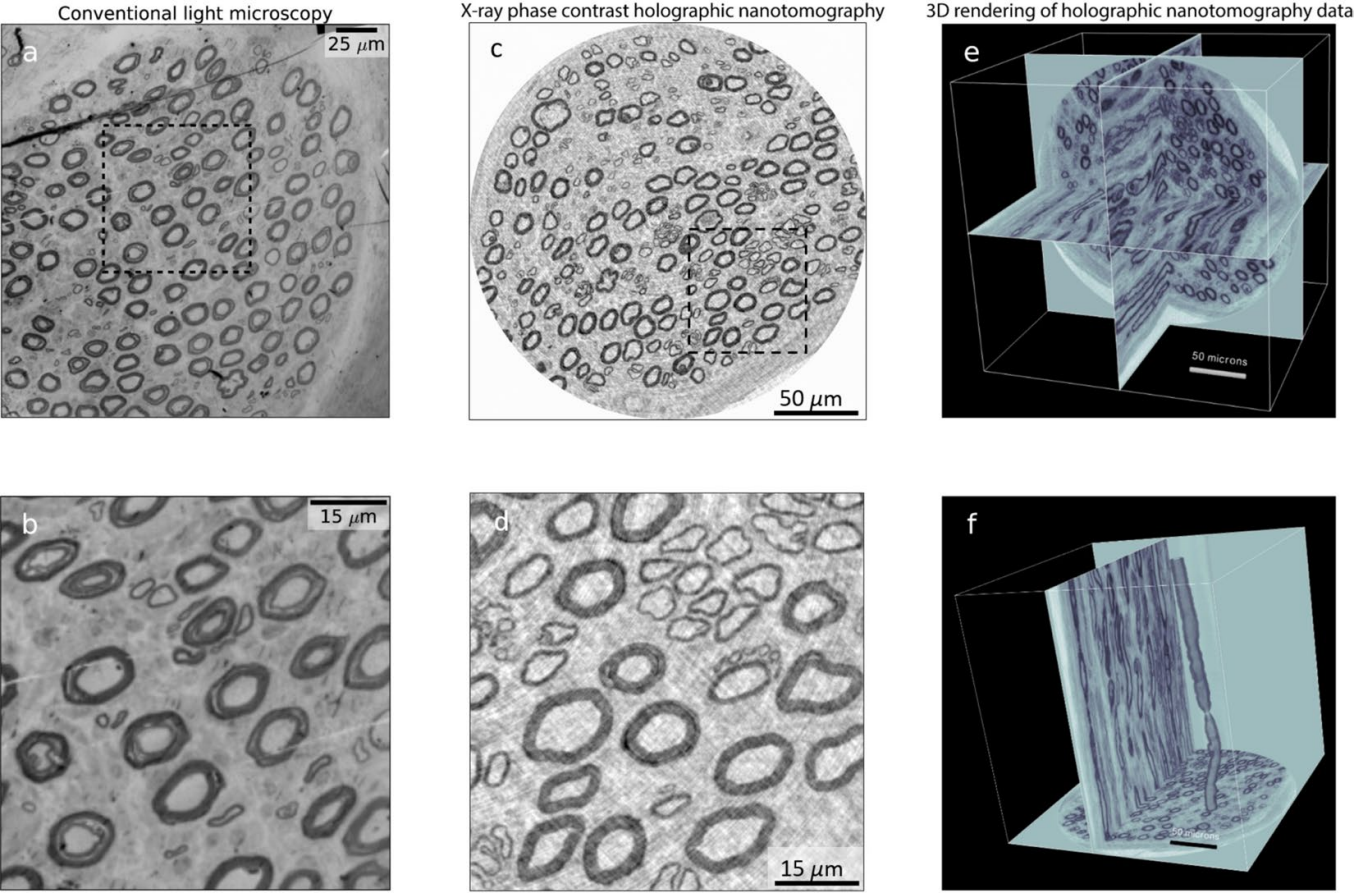
Comparison between conventional light microscopy and X-ray phase contrast holographic nanotomography. (**a**) Light microscopy image of a physical slice of a nerve sample. (**b**) Enlargement of the sample area marked in (**a**). (**c**) Digital slice from a 3D volume imaged by X-ray phase contrast holographic nanotomography. **(d)** Enlargement of area marked in (**c**). (**e**) Visualization of three perpendicular planes of the X-ray data volume. (**f**) Visualization of two perpendicular planes and a 3D rendering of a single axon with a node of Ranvier visible.

Epon embedded nerve samples were imaged at the nano-imaging beamline ID16A-NI at the European Synchrotron Radiation Facility (ESRF). An X-ray beam of 17 keV photon energy was focused with an elliptical Kirkpatrick-Baez optics system as described by da Silva *et al*.^22^ and Mokso *et al*.^23^. Details about the custom made sample positioning and manipulation stage is described by Villar *et al*.^24^. Tomographic scans at four different focus-to-sample distances (52.6 mm, 54.9 mm, 63.9 mm and 82.7 mm, respectively) have been acquired for each sample, while keeping a constant distance between the focus point and the detector of 337.3 mm. In each tomography scan 1500 projections were recorded over 180 degrees, with an exposure time of 0.5 s and an angle of 0.12 degree per frame. Total duration for each tomography scan was 2 hours and 10 minutes. For each rotational angle, the four recorded projections were combined to obtain a phase map which was then used for tomographic reconstruction^25,26^. The effective pixel size was 130 nm, resulting in a reconstructed volume of 2048 × 2048 × 2048 voxels of isotropic voxels, covering a 266 × 266 × 266 μm^3^ field of view, which fits well with the size of the nerve fascicles containing the axons. We have evaluated the spatial resolution by measuring the full width at half maximum of the analytical derivative of a hyperbolic tangent function fitted to the grey values corresponding to the transition between myelinated axons and the surrounding tissue. The average value of 12 measurements at different locations was 278 nm. These measurements were applied to the data illustrated in Fig. 1 panels 1c and 1d. The total radiation dose delivered to the sample during the four tomography scans was 1.2 MGy.

### Segmentation

**Figure 2:**
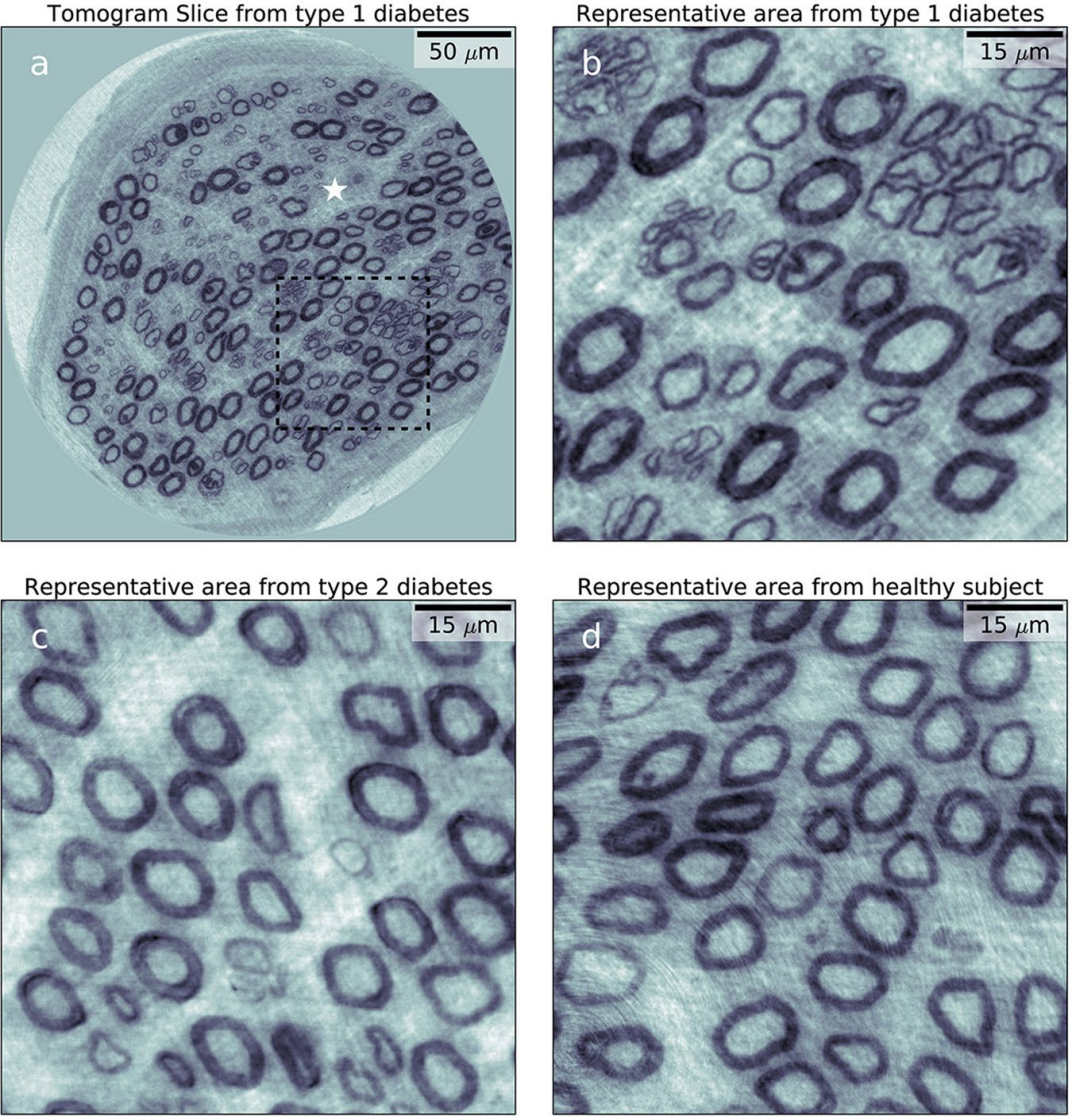
Representative sample areas from subject with type 1 diabetes, with type 2 diabetes and a healthy subject. (**a**) Full view of digital slice from subject with type 1 diabetes. The star marks a blood vessel containing a red blood cell. (**b**) Enlargement of the selected representative area marked in (**a**). (**c**) Representative area of subject with type 2 diabetes. (**d**) Representative area of healthy subject.

For quantitative analysis and visualization, the axons illustrated in Fig. 1(f) and 2 were segmented by fitting surfaces to the CT data using a semi-automated approach. In the obtained volumetric data, the nerve fibres are aligned with the z direction, and myelin sheaths appear circular in the x-y slices through the volume. This allowed us to perform a slice-wise segmentation of an individual fibre by aligning a closed curve with the periphery of the nerve. For this, the circle is initialized around the nerve, and is automatically moved to the boundary of the myelin, utilizing the fact that myelin appears dark compared to the background. For segmenting a whole fibre, the curves are automatically propagated through the volume, such that the surface moves only slightly between the slices, and is in every slice attracted to the boundary of the myelin layer. The approach is similar to the method from Dahl *et al*.^27^. Through inspection, we ensured that nerves are precisely segmented. We performed a few manual corrections when needed.

### Ethics

All samples were collected according to the Swedish ethics regulations. The project was approved by the Regional Ethical Review Board at Lund University (LU 508-03) which included detailed morphological analyses of the nerve samples with various techniques. The nerve biopsy procedure was approved by the Regional Ethical Review Board as part of a larger research project. Informed consent was obtained from all participants after oral and written information. All observations were performed on coded samples.

## Results

### Comparison of microscope images

Histological technique with light and electron microscopy is the standard imaging technique for neuropathology, where thin sections are cut from the sample or teased nerve fibres are harvested for evaluation in a microscope^28^. In this study, we evaluated the feasibility of using X-ray phase contrast holographic nanotomography to investigate the 3D micro-morphology of a human peripheral nerve from single patients with type 1 and type 2 diabetes as well as from a healthy subject.

Figure 1 displays a qualitative comparison between images obtained with conventional histology with a light microscope (Fig. 1: Panels 1a and 1b) and X-ray phase contrast holographic nanotomography (Fig. 1: Panels 1c and 1d). Panel 1c displays a single slice of the tomographic X-ray data for direct comparison with the microscopic image in panel 1a, where a thin slice of tissue has been imaged with visible light (i.e. light microscopy). Panels 1b and 1d are magnifications of Panels 1a and 1c, respectively. While the quality of images obtained by light microscope is better than that of X-ray phase contrast holographic nanotomography, these images are limited to two dimensions. The volumetric information obtained from X-ray phase contrast holographic nanotomography allows for slicing the same sample in arbitrary direction (as illustrated in Fig. 1: Panel 1e) and creation of 3D renderings (as illustrated in Fig. 1: Panel 1 f). In addition, browsing through the stack of slices allows for much better identification of morphological features [see Supplementary Figs. 1 (S1) and 2 (S2)]. A node of Ranvier with the adjacent paranode is displayed in three perpendicular virtual sections in Supplementary Fig. 1 (S1). Schmidt-Lantermans incisure is envisioned in a similar manner in Supplementary Fig. 2 (S2).

### Characterization of structures in two patients with type 1 and 2 diabetes and in a healthy subject

Figure 2 displays representative pictures from the X-ray phase contrast holographic nanotomography volumes acquired of samples from two patients with diabetes type 1 (Panel 2a and 2b), and type 2 (Panel 2c) as well as from a healthy subject (Panel 2d).

#### Peripheral nerve from a patient with type 1 diabetes

The specimen of the posterior interosseous nerve from the patient with long-term type 1 diabetes showed, based on a qualitative assessment, generally a low density of large myelinated nerve fibres (Panels 2a and 2b) relative to the healthy subject (Panel 2d) in agreement with previous work^29^. Smaller myelinated nerve fibres, mainly interpreted as regenerative clusters, were detected in-between the larger myelinated nerve fibres (Panels 2a and 2b). A blood vessel, with less contrast, but with a red blood corpuscle visible, was visualized in the cross section (indicated by a star in Panel 2a). Nodes of Ranvier and Schmidt-Lanterman incisures were also noted [see Supplementary Figs. 1 (S1) and 2 (S2), respectively].

#### Peripheral nerve from a patient with type 2 diabetes

The posterior interosseous nerve specimen of the female patient with type 2 diabetes with a shorter duration of diabetes showed, based on a qualitative evaluation, generally a similar density of large myelinated nerve fibres (Panel 2c) as in the healthy subject (Panel 2d). A smaller number of small myelinated nerve fibres, interpreted as regenerative nerve fibres, were observed in-between the larger myelinated nerve fibres (not shown).

#### Peripheral nerve from a healthy subject

The specimen of the posterior interosseous nerve from the healthy subject showed overall a regular and dense distribution of large myelinated nerve fibres (Panel 2d), where the axons and the osmium stained myelin sheaths could clearly be visualized. Medium-sized and small myelinated nerve fibres were also observed in-between the larger myelinated nerve fibres. The structures mentioned above were clearly seen in the 3D volume.

### Extraction of morphological data

**Figure 3:**
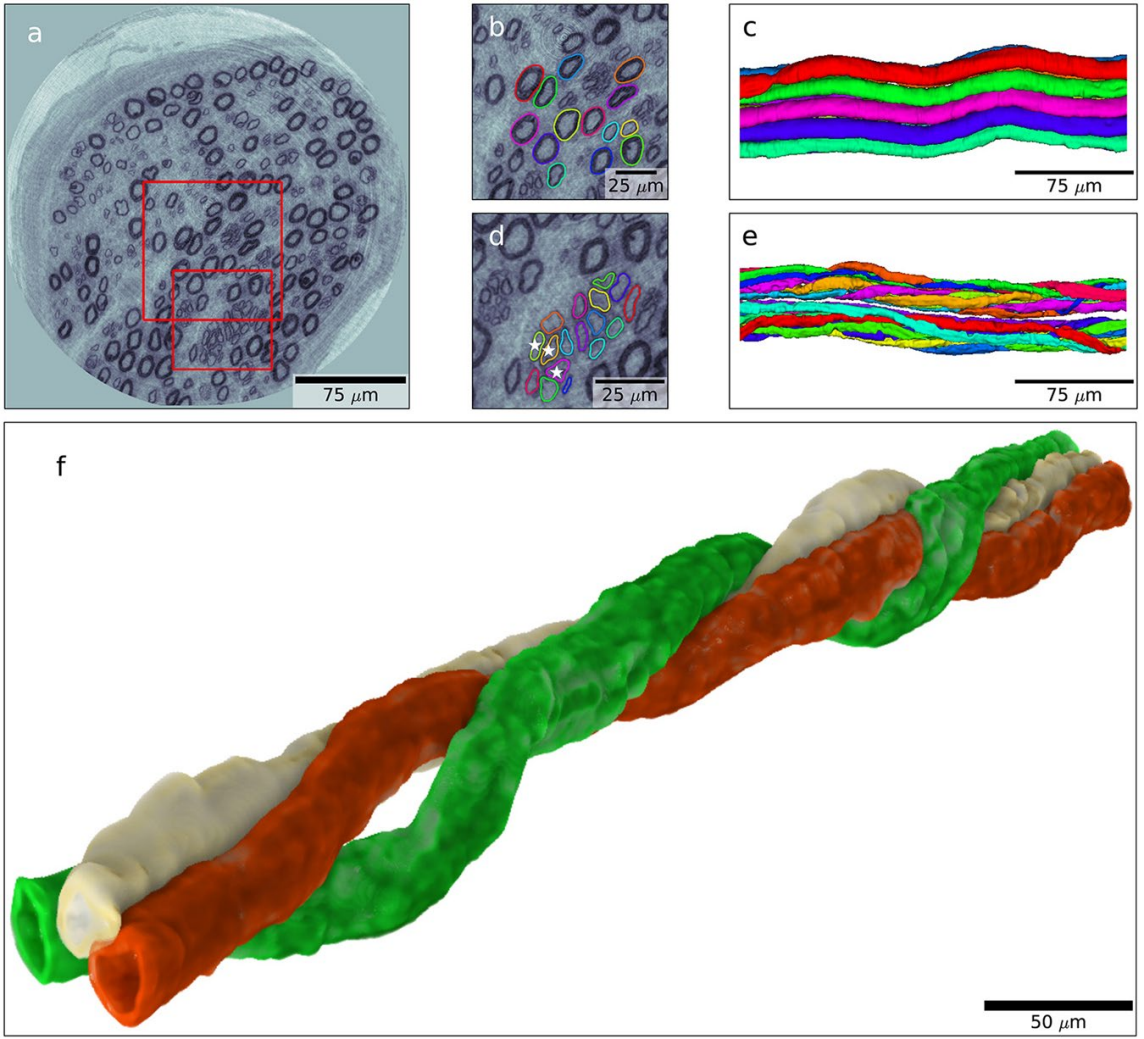
Examples of semi-automatic segmentation. (**a**) Tomographic slice through the data volume of the subject with type 1 diabetes. (**b**) Enlarged area marked by a large red square in (**a**). Coloured rings mark the segmented nerves calculated by our semi-automated algorithm. (**c**) 3D rendering of the segmented nerves marked in (**b**). (**d**) Enlargement of area marked by small red square in (**a**). Clusters of regenerated axons are segmented by our semi-automated algorithm. (**e**) 3D rendering of the segmented nerves clusters segmented in (**d**). (**f**) 3D volume rendering of the three axons marked by stars in (**d**). Note how one axon twists around the other two.

The semi-automatic segmentation algorithm described in the methods section was applied to the volumetric X-ray phase contrast holographic nanotomography data. Figure 3 displays an example of such segmentation and data extraction on a sample from the patient with type 1 diabetes. Panel 3a displays a single slice of data, where a subset of the larger myelinated nerve fibres has been selected for segmentation. The segmentation algorithm is then applied to the volume, and the resulting segmented fibres can be analysed and visualized (Panels 3b and 3c). The small myelinated fibres, identified as regenerative clusters and visualized in Panels 3d and 3e, reveal an intertwining of the axons [further visualized in panel 3 f and in the Supplementary movie (S3)], which is completely different from the appearance of normal myelinated nerve fibres with the waveform in the same fascicle. Note that in panel 3e, two different regenerative clusters are segmented, separated by a thin layer of connective tissue. Thus, there was no intertwining between these two clusters as can be clearly seen in Supplementary movie S3.

### Regeneration event

**Figure 4:**
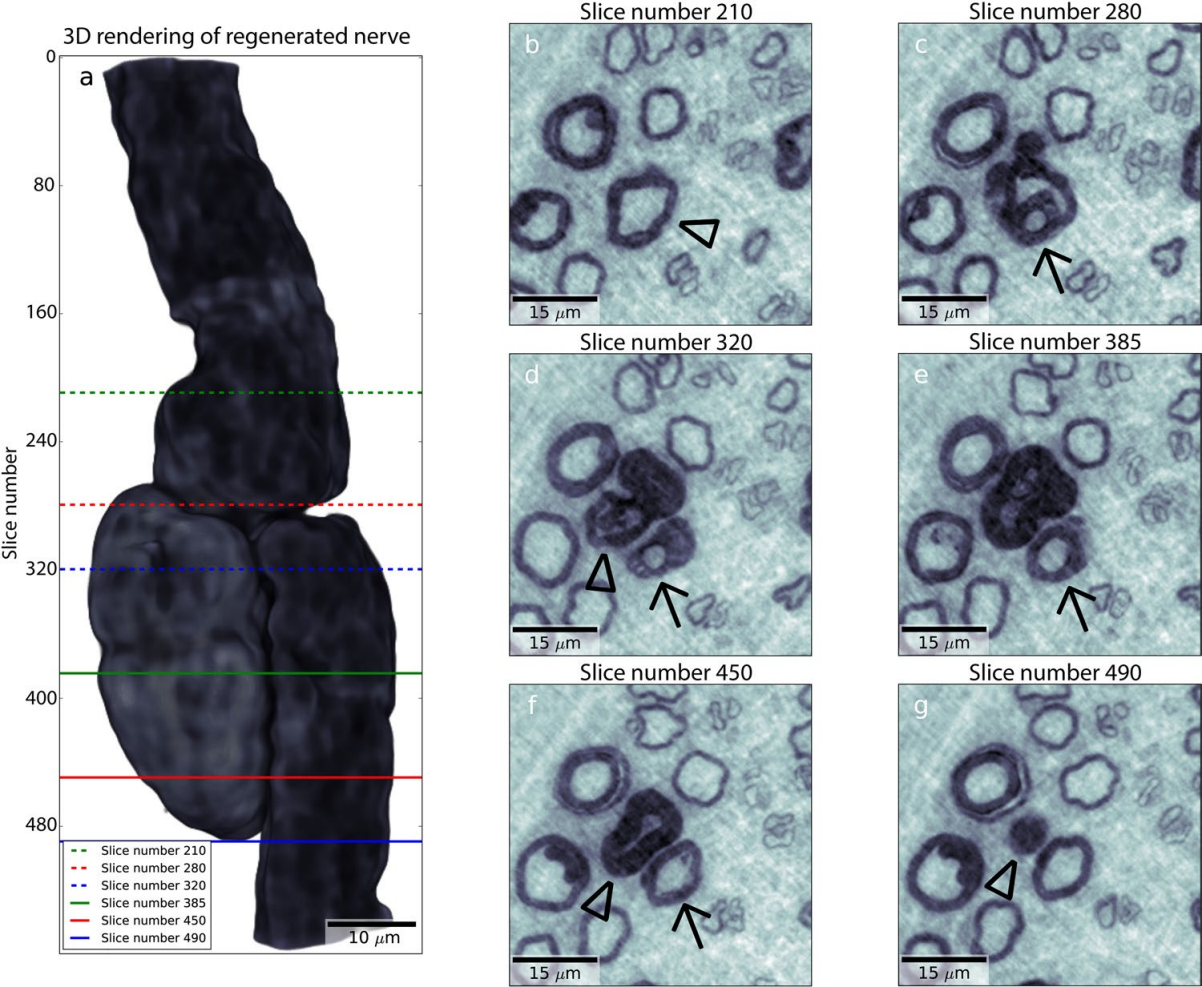
Visualization of regenerative event. (**a**) 3D rendering showing only the abnormal axon. The regeneration event is visualized in panels (**b**–**g**), indicated by lines in panel (a). Arrow: regenerating axon. Arrowhead: original axon.

In the specimen from the patient with type 1 diabetes, a microanatomical structure, interpreted as a regenerative event, was found (Fig. 4). A normal myelinated nerve fibre was found together with a node of Ranvier, but a bulb of myelin with a “dead end” was observed attached to one of the paranodes (Panel 4a). We interpret the structure to be a degenerated myelinated nerve fibre, with the myelin bulb still attached, from which a new axon has sprouted and become myelinated. Cross sections from the tomography are shown in Fig. 4 panels (b-g). In slice 210 (panel 4b), a normal axon is found (arrowhead). A new regenerating axon with myelin is formed in slice 280 (panel 4c; arrow). The regenerating axon (arrow) separates from the degenerating segment of the original axon (arrowhead) in slice 320 (panel 4d) and mature in slice 385 (panel 4e; arrow). In slice 450 (panel 4 f), the new regenerating axon with myelin (arrow) is visible next to the degenerated axon (arrowhead), which is still seen in slice 490 (panel 4 g; arrowhead). A video of all the slices in the volume presented in Fig. 4 is available as supplementary information (Supplementary Fig S4).

In Supplementary Fig. 5 (S5) an electron microscopical image is added to show two different Schwann cells with a similar feature as presented in Panel 4 g (slice 490).

## Discussion

The present study has demonstrated the potential of and feasibility of using X-ray holographic nanotomography to visualize the cellular and subcellular structures of single human peripheral nerves. Quantitative three-dimensional (3D) structural data, which can be related to conventional two-dimensional images, is important to further understand the pathophysiology and the detailed cellular networks of peripheral nerves in diabetic neuropathy. By the use of osmium tetroxide staining, by which the present specimens were originally stained for conventional morphometry^19,20,29^, it is possible to distinguish between the axon and the surrounding myelin sheath as well as to identify substructures of the nerve fibres, such as the nodes of Ranvier, with their paranodes and paranodal remodelling^30^, and Schmidt-Lanterman incisures^17^, with the goal to evaluate structural alterations in diabetic neuropathy. In view of the potential use of different imaging techniques, such as synchrotron based X-ray microscopy^10,11,17,18,31^ and other microscopy techniques^5–9,32^, we were able in the present study with a limited number of specimens to show detailed structures, as evaluated by a qualitative assessment, of the peripheral nerve, using the biopsies from the posterior interosseous nerve in the upper extremity in one healthy human subject and in two patients with type 1 and 2 diabetes^19,20^. Structural alterations in the sural nerve in type 1 and 2 diabetes, characterized by demyelination, degeneration and regeneration events of myelinated nerve fibre with changes in fibre density^33^ and with remyelination remodelling found at the paranodal region^30^, have previously been studied and also quantified by conventional light and electron microscopical techniques, which only allows limited dimensional analyses^19,21,28,34^. We showed degeneration in the single patient with type 1 diabetes, which is in agreement with an earlier study on the same nerve biopsies^29^, but due to the small number of nerve specimens it was not relevant to make any quantitative measurements and analysis of the morphometry here. Interestingly, analyses of single specimens in post-mortem brains from a few patients with schizophrenia have also been utilized in detection of certain structural changes at high resolution by the use of X-ray nanotomography^35,36^. As in previously published work by Bartels *et al*.^17^ from mouse sciatic nerve, the 3D details of human myelinated nerve fibres, such as nodes of Ranvier, including the paranode, and Schmidt-Lanterman incisures, were observed in the present study. Due to the contrast discrepancies between the osmium stained myelinated nerve fibres and the intraneural blood vessels^28,37^, it was not possible to perform a 3D structure of the intraneural microvasculature with the segmentation technique. However, we could visualize one of the blood vessels, including blood corpuscles, with thickened basement membrane in the specimen from the patient with type 1 diabetes. To further characterize intraneural microvasculature in the future, specimens without osmium staining should be used.

Interestingly, we were able to show the detailed 3D structure of the regenerative clusters with intertwining of the nerve fibres; i.e. “misdirected” outgrowing nerve fibres, in the patient with type 1 diabetes in contrast to the normal appearance of the myelinated wave-formed nerve fibres in the fascicle found in the healthy subject. Regenerative clusters in 2D images have earlier been described, but the intertwining can only be observed with 3D imaging techniques. To our knowledge such an intertwining has never earlier been depicted, where it even in the specimen was possible to define two adjacent clusters with thin connective tissue in-between. Such a detailed 3D visualization of pathological alterations at the cellular level in diabetic neuropathy is not possible with conventional imaging techniques, including micro-CT, for describing 3D structure.

A remarkable novel finding was the observation in the patient with type 1 diabetes, where a myelinated nerve fibre with its paranodes and node of Ranvier, and with an attached myelin bulb, representing remodelling, was found. We interpret this finding as a degenerating myelinated nerve fibre, with a remaining part observed as a bulb, and a new outgrowing, and remyelinated, axon originated from the nearby paranode. This structure has never been described in 3D or interpreted correctly with conventional light or electron microscopical techniques, as shown in Supplementary Fig. 5 (S5), or even with micro-CT; thus, demonstrating the potential of the present technique. Furthermore, with the segmentation at hand, a number of quantitative parameters can be extracted for statistical analysis, but require a larger number of nerve biopsies for analyses of the detailed morphometry. Even the thickness of the myelin sheath can be extracted by the present technique with statistical analyses when a larger number of samples from healthy subjects and patients with diabetes are available (see Panel 1f). Characterization has also been performed in a number of other tissues by the use of X-ray computed micro- and nanotomography indicating the benefit of the techniques^38–40^.

There are a variety of other techniques that have been used to visualize nerve fibres in different, mainly experimental, projects, where several published papers have used confocal microscopy, sometimes combined with electron microscopy^41–44^. Scanning in z-direction enables 3D structures of various components of the peripheral nerve or of other samples from the nervous systems. Scanning electron microscopy is another alternative for visualization, although time consuming, where it is possible to reconstruct 3-D structure through sections^45^ with image stack covering 60 ×150–200 um from 0.5 ×0.5 mm specimens. However, it is not with such a technique possible to cover a bunch of axons; thus, not possible to show long healthy and particularly the interwoven regenerating nerve fibres from the regenerating clusters from larger specimens as in the present study although a 3D image of a single axons from Ness *et al*. is similar as ours^45^. The techniques used by Ness and Ohno^43,45^ require treatment of the tissue with heavy metals, which may be a disadvantage in analyses. None of the mentioned articles describe non-myelinated axons, or newly myelinated regenerating nerve fibres as regenerative clusters described in the present nerve biopsies. Furthermore, such a visualization is not possible with described micro-CT techniques due to the lack of resolution^5–9^. With the present technique, we can also visualize nodes of Ranviers and Schmidt-Lantermans incisures with high resolution together with details of intraneural blood vessels. Furthermore, Schain *et al*. describe myelinated axons with a parallel structure from confocal microscopy in movies in agreement with present images^44^. However, none of the previous published literature describe in movies newly regenerating axons (“a new-born axon”), which is possible with the present synchrotron technique. To conclude, even if a variety of other techniques are available to visualize nerve fibres, the strength with the present synchrotron technique is that we can demonstrate the detailed structure of myelinated healthy and regenerating axons over longer distances, both longitudinally and cross-sectioned, with a resolution in the sub-micron range and with the potential in the future to statistically analyse morphometry in a larger number of samples.

This is the first study to use X-ray phase contrast holographic nanotomography to visualize human myelinated nerve fibres and their subcomponents in 3D. We are able to present novel findings on qualitative differences between a healthy subject and two patients with type 1 and 2 diabetes, in which the 3D structure of regenerative clusters with twisted nerve fibres and a novel detailed 3D view of a sprouted axon were visualized. We believe that, based on this feasibility study, X-ray phase contrast holographic nanotomography in the future can provide new insights into the pathophysiology of not only diabetic neuropathy, but also of other pathological conditions.

## Supplementary information

Supplementary Information.

Supplementary Figure 3 - Video

Supplementary Figure 4 - Video

## Data Availability

The material consists of nerve biopsies from three humans taken after ethical permission from the local ethical committee. Written informed consent was provided by the patients. Public access to the data is restricted by the ethical committee securing that no data should be transformed to any third party with the risk of interfering with the identity of the patients.
