# Supplementary material for: Three-dimensional architecture of human diabetic peripheral nerves revealed by X-ray phase contrast holographic nanotomography
Source: Sci Rep. 2020 May 5;10:7592. doi: 10.1038/s41598-020-64430-5 (PMC7200696; doi:10.1038/s41598-020-64430-5)
Supplement: Supplementary file 1 — Supplementary Information. [file 41598_2020_64430_MOESM1_ESM.docx]

**Supplementary information**

**Three-dimensional architecture of human diabetic peripheral nerves revealed by X-ray phase contrast holographic nanotomography**

Lars B Dahlin^1,2†^, Kristian R Rix^3†^, Vedrana A Dahl^4^, Anders B Dahl^4^, Janus N Jensen^4^, Peter Cloetens^5^, Alexandra Pacureanu^5^, Simin Mohseni^6^, Niels OB Thomsen^2^ and Martin Bech^7*^

†=shared first authorship, *=corresponding author

From the ^1^Department of Translational Medicine – Hand Surgery, Lund University, Sweden; ^2^ Department of Hand Surgery, Skåne University Hospital, Malmö, Sweden; ^3^Niels Bohr Institute, Copenhagen University, Copenhagen, Denmark; ^4^Department of Applied Mathematics and Computer Science, Technical University of Denmark, Lyngby, Denmark; ^5^European Synchrotron Radiation Facility, Grenoble, France; ^6^Department of Clinical and Experimental Medicine, Linköping University, Linköping, Sweden; ^7^Department of Medical Radiation Physics, Clinical Sciences Lund, Lund University, Lund, Sweden;

Corresponding author: martin.bech@med.lu.se

Supplementary information

Supplementary figure 1.


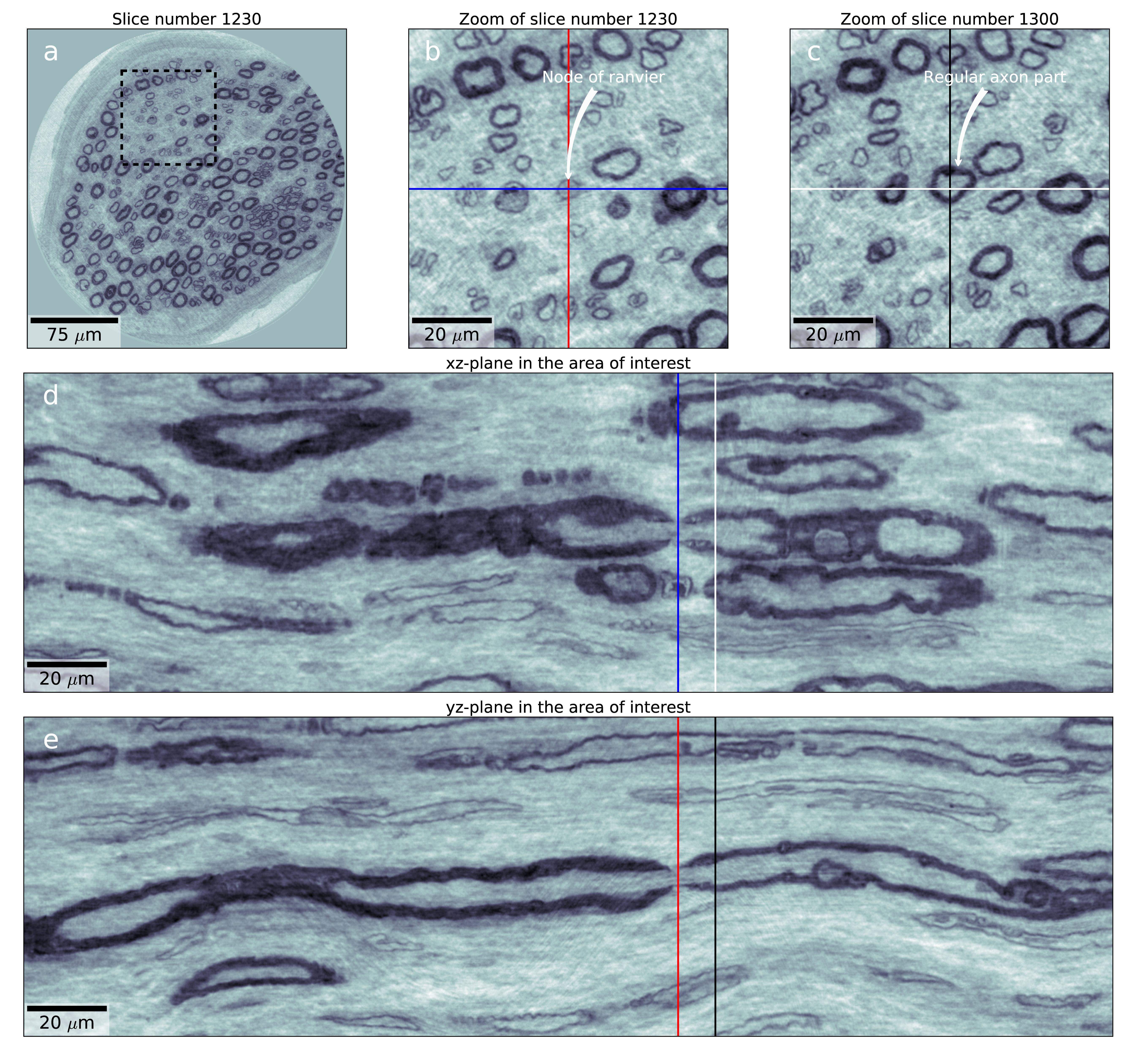


Supplementary figure 1. Illustration of a node of Ranvier. a) Digital slice from data volume containing a node of Ranvier. b) Enlargement of the area marked in (a) with the node of Ranvier marked by an arrow. c) Slice parallel to that displayed in (b), but separated by 9.1μm. d) The xz-plane perpendicular to the slices in panels (b) and (c). Two colored lines show where the planes intersect. e) The yz-plane perpendicular to the slices in panels (b), (c) and (d). Two colored lines show where the plane intersect the slices in panels (b) and (c).

Supplementary figure 2.


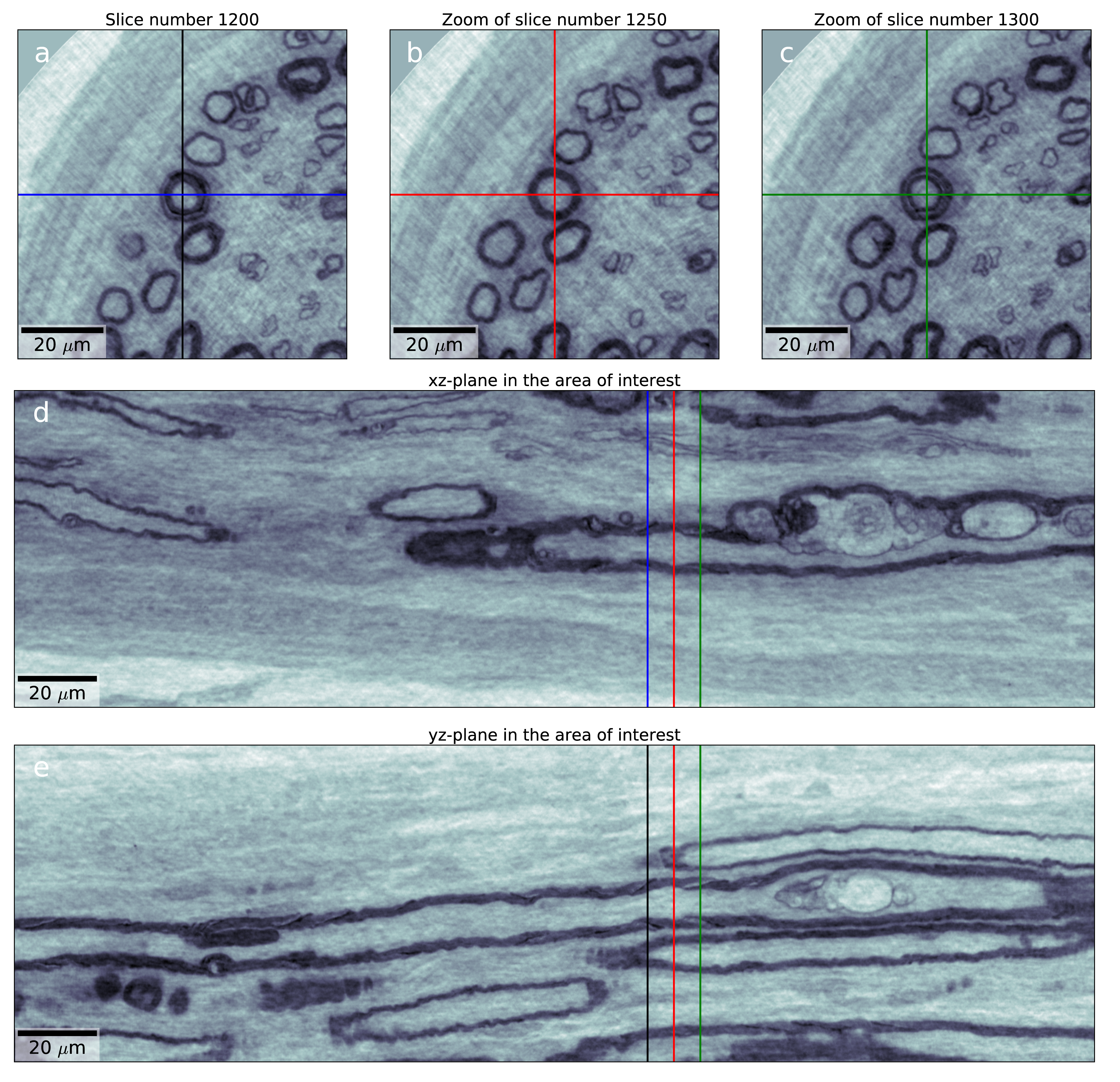


Supplementary figure 2. Illustration of a Schmidt-Lantermans incisure. a) Digital slice 1200 from data volume containing Schmidt-Lantermans incisure. The nerve fiber containing the Schmidt-Lantermans incisure is marked by the intersection of two colored lines. b) and c) Slices parallel to (a) separated by 6.5μm. d) The xz-plane perpendicular on the slices in panels (a), (b) and (c). Three colored lines show where the plane intersects the slices in panels (a), (b) and (c). e) The yz-plane perpendicular to the slices in panels (a), (b), (c) and (d).

Supplementary figure 3. Video


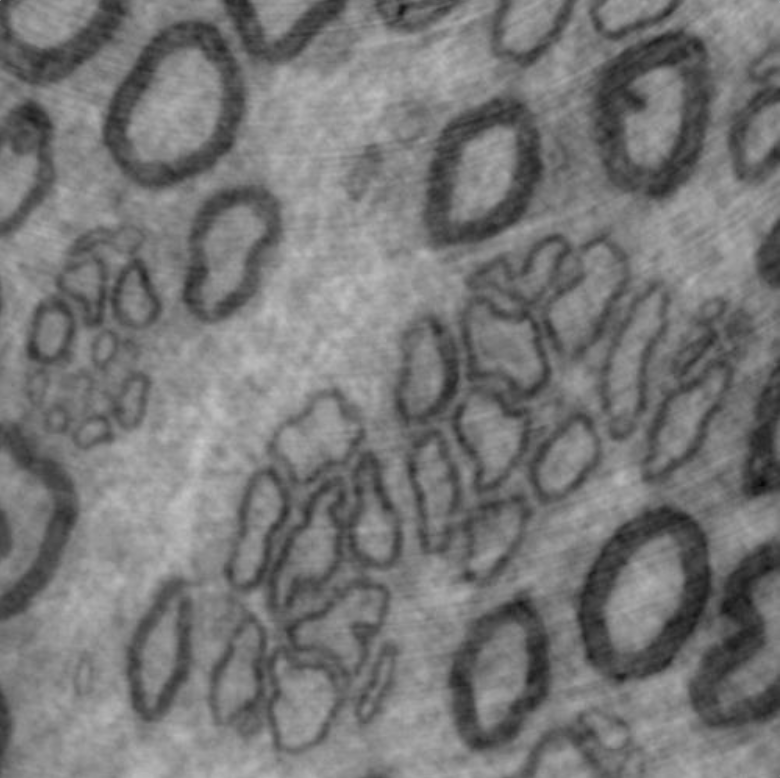


Supplementary figure 3. Video illustrating the intertwining axons in the regenerating clusters. Voxel size 130 nm x 130 nm x 130 nm.

Supplementary figure 4. Video


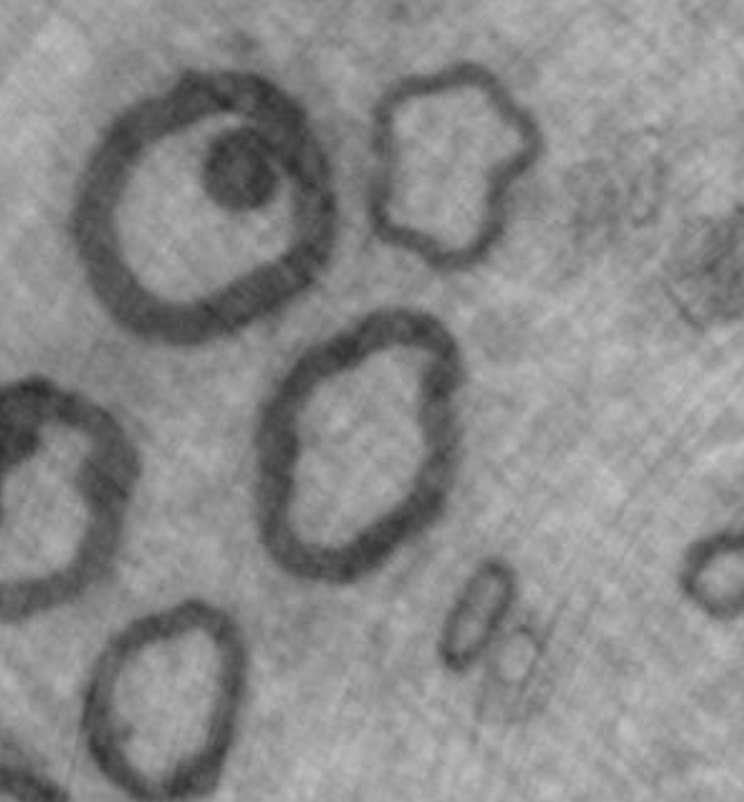


Supplementary figure 4. Video illustrating the regeneration event described in figure 4.

The most interesting biological events appear at the following times:

At time 0-10 second (at the central part of the picture) there is a normal myelinated axon.

At second 11; two axons are separated from each other by myelin.

At second 13; a small axon is identified to the lower right with some myelin around, and a big axon to the left of it. Here a small myelinated axon is “born”.

At second 13.5; a node in the small axon appears.

At second 13.5-15; the small axon is getting more myelin.

At second 17, a normal myelinated axon of the small one,

At the end of second 23, the small axon is now a very nicely myelinated, and the larger axon starts to degenerate.

At second 24; only some myelin is left of the larger axon.

At second 26; the larger axon and its myelin is gone, and does not appear in the rest of the movie.

Supplementary figure 5.


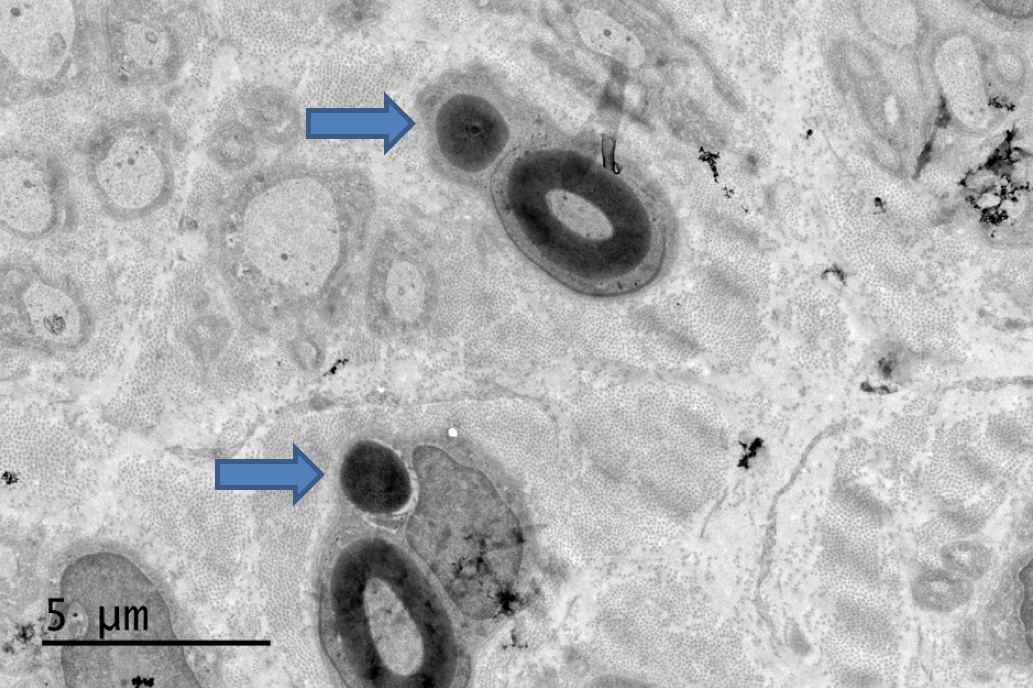


Supplementary figure 5. Electron microscopy image showing two normal myelinated axons, each with a Schwann cell. The Schwann cells also include myelin structures (arrows) that are unrelated to the axons. This is similar to that observed in Figure 4g (slice 490) and in the Supplementary video 4 at seconds 24 - 26, where the degenerated myelinated axon is sealed.
